# The League of Mercy at Marlborough House

**Published:** 1902-06-07

**Authors:** 


### THE LEAGUE OF MERCY AT MARLBOROUGH HOUSE.

### RECEPTION BY THE PRINCE AND PRINCESS OF WALES.

Under any circumstances the reception on Monday at Marlborough House by the Prince and Princess of Wales of several hundred members of the League of Mercy would have been an extremely interesting event. But owing to the lovely weather which prevailed on the occasion, and set off to advantage the charming costumes, the scene in the gardens was one of great brilliance. There was lacking no element calculated to render the proceedings delightful to the guests.

The time fixed for the ceremony was half-past three, and soon after the clock struck three, carriages began to draw up at the gates, which were opened at a quarter past. The company were received, on behalf of the Prince and Princess of Wales, by Lieut.-Colonel the Hon. Sir William Carington, Comptroller of the Prince's Household, who explained that their Royal Highnesses would be detained in the House of Lords until five o'clock, owing to their anxiety to hear the Prime Minister communicate to the Chamber the terms of the Boer surrender. In the interval, the guests strolled about the beautiful grounds, sat under the trees, and listened to the performance of an excellent musical programme by the band of the Royal Marine Light Infantry, from Portsmouth, conducted by Lieutenant C. J. Miller, or partook of refreshments which were served in a tent. Of course, the more general topic of conversation was the question of the day, and the conclusion of peace, no doubt, added to the happinsss of the hour. Next only in importance to the guests was the work of the League itself. It was pleasing to hear the satisfaction expressed at the progress which the movement has made in three years, though, as an enthusiastic lady said, "with 60 presidents, 50 lady presidents, and ever so many more vice-presidents, we ought, instead of £14,000, to have subscribed £20,000 to the London hospitals." The decorations of the order for conspicuous service which were placed ready for bestowal in neat cases, were carefully examined by many, and excited considerable admiration. They are in the form of the Geneva Cross in deep red enamel upon gold, having in the centre a tiny figure representing Mercy. Some of the young ladies who expressed regret that it was not their lot to receive one, were consoled by the official assurance that Coronation year would afford an excellent opportunity to put into execution the resolution to earn the badge, if they were so minded, though it requires some years of arduous labour to secure it.

Punctually at five o'clock the Prince and Princess of Wales arrived and took up their position beneath an awning erected on the south side of the lawn. Their presence was proclaimed to the audience in the gardens by the playing of the National Anthem, which was followed by "Rule Britannia," and the whole of the company were quickly grouped in the vicinity of the awning. Their Royal Highnesses who are respectively Grand President and Lady Grand President of the League—the King being Patron and Sovereign—were accompanied by Prince Edward, Prince Albert, and Princess Victoria of Wales. The Princess of Wales, wearing a dress of cream voile trimmed with Irish lace, her tulle toque being adorned with pink roses. In attendance were Lady Eva Dugdale and Lord Wenlock, there being also present Colonel Sir William Carington, and the honorary officers of the Order, Colonel Knollys, Lord Wolverton, Dr. W. J. Collins, Mr. J Harrison, and Sir Henry Burdett, K.C.B.

Before proceeding to distribute the badges, the Prince of Wales said: "Ladies and Gentlemen,—I wish to tell you how very pleased both the Princess and I are to see you all here this afternoon, and we wish to thank you for all the work and the trouble you have had with regard to the League of Mercy." The decorations were next handed by Colonel Knollys to the Grand President, who first affixed them to the dress of each of the lady recipients, and then to the coat of each of the gentlemen, the Lady Grand President shaking hands with all of those upon whom the order had been conferred. The following thirteen ladies received the order:—

The Viscountess Parker (St. George's, Hanover Square); Lady Farquhar (West Marylebone), The Hon. Mrs. Halford (Westminster), Lady Maclean (Hythe), Lady Faudel-Phillips (Hertford), Lady Treloar (Croydon), Mrs. Nettleton Balme (Sevenoaks), Mrs. Boscawen (Tonbridge), Mrs. R. L. Harrison (Fulham), Mrs. Edwin Hughes (Woolwich), Mrs. Buxton Morrish (Lambeth Norwood), Mrs. Schloss (South Kensington), Miss Mary Thompson (Croydon). Lady Pirbright (Guildford) and Mrs. Charles Davis (West Marylebone) were also awarded the decoration, but were unable to attend to receive it in person.

The following sixteen gentlemen received the order:—The Marquess Camden (Tonbridge), The Earl of Mansfield (North St. Pancras), Col. Owen (Fulham), Col. Gorham (South Kensington), Col. Hamilton (St. George's Hanover Square), Capt. Speer (Epsom), Lieut. A. H. Tarleton, R.N. (Deptford), Mr. R. C. Antrobus (St. George's, Hanover Square), Mr. A. H. Baker (Sevenoaks), Mr. P. Cremieu Javal (City of London), Mr. T. C. Dewey (Sevenoaks), Dr. James Harper (South Kensington), Alderman John Harris (Whitechapel), Mr. T. B. Jobson (Ashford, Kent), Mr. J. C. Marshall (East Marylebone), Mr. H. J. Palmer (East Marylebone).

The distribution of the decorations having finished, the remainder of the guests, exceeding 700 in number, passed by the Prince and Princess of Wales, and their names in all cases having been announced, were presented to their Royal Highnesses. They included the Presidents and Lady Presidents of the following districts:—Battersea and Clapham, Lord and Lady Battersea; Bethnal Green, Sir M. M. Bhownaggree, K.C.I.E. M.P., and Miss Harrington; Camberwell (Peckham), Mr. Goddard Clarke,; Croydon, Alderman Sir W. P, and Lady Treloar; Deptford, Lieut. A. H. Tarleton and Lady Florence Pelham Clinton; Finsbury (Holborn), Mrs. Dibdin; Finsbury (East), Mr. and Mrs. J. Allen Baker; Fulham, Col. A. A. Owen and Mrs. R. L. Harrison; Greenwich, Mrs. Stidolph; Chelsea, Lord James of Hereford and the Countess Cadogan; Hackney (North), Mr. Walter Johnson (Mayor of Hackney) and Mrs. Johnson; Hackney (South), Mrs. Green; Hammersmith, Mr. W. J. Bull, M.P., and Mrs. Brandon; Hampstead, Mrs. Withman; Islington (West), Mrs. Hodson; Kensington (South), His Grace the Duke of Argyll, K.T.; Lambeth (Brixton), Mr. W. and Miss Hayden; Lambeth (North), Col. Ford; Lambeth (Norwood), Col. Campbell and Mrs. Buxton Morrish; Lewisham, the Earl and Countess of Dartmouth · Marylebone (East), Mrs. Boulnois; Marylebone (West), Lord Farquhar, K.C.V.O., and Lady Farquhar; Newington (West), Col. John Davis, A.D.C., and Lady Llangattock; Paddington (South), Lady Burdett; St. George's Hanover Square, Mr. R. C. Antrobus and Countess Grosvenor; St. Pancras (North), Earl of Mansfield and Dowager Lady Lamington; St. Pancras (East), Mr. T. H. W. and Mrs. Idris; St. Pancras (South), Col. R. Edis; Southwark (Rotherhithe), Mr. Ambrose Pomeroy, Esq.; Mile End, Mr. B. S. Straus, Esq, and Mrs. Sidney Straus; Whitechapel, Alderman John Harris and Mrs. Lawson; Woolwich, Col. Edwin and Mrs. Hughes; Westminster, Mrs. Seymour Corkran; Colchester, Sir W. D. Pearson, Bart, M.P. and Mrs.

Sanders; Epping, Col. Lockwood, M.P., and Mrs. Lockwood; Harwich, Mr. James Round, M.P., and Mrs. Gurdon-Robow; Maldon, Major-General Sir H. Ewart, K.C.V.O., K.C.B., and Lady Evelyn Ewart; Romford, Mr. F. Green and Mrs. McIntosh; Walthamstow, Mr. A. Lister Harrison and Lady Loucha Warner; Hertford, Mr. Ernest Pearson and Lady Faudel-Phillips; Ashford, Lord Medway; Dartford, the Right. Hon. Sir W. Hart-Dyke, Bart., M.P.; Faversham, Lord Harris, G.C.S.I., G.C.I.E., and Lady Harris; Hythe, Sir Fitzroy Maclean, Bart., C.B., and Lady Maclean; Isle of Thanet, Sir Sebag-Montefiore; Medway, Mr. F. S. W. Cornwallis and Viscountess Torrington; Sevenoaks, Mr. E. A. Hambro; Tonbridge, the Marquess Camden and Mrs. Boscawen; Brentford, Mr. Montagu Sharpe; Chertsey, Mr. H. C. Leigh-Bennett, M.P.; Wimbledon, Mr. Tyrrell Giles, M.P., and Mrs. Tyrrel Giles; Eastbourne, Mr. H. W. Keay and the Hon. Mrs. Freeman-Thomas.

In addition to these were a large number of vice-presidents, as well as members, the march past not being concluded until nearly 6 o'clock, the Prince and Princess shaking hands with each. There was no speaking, and as soon as the reception had terminated, their Royal Highnesses took tea in their marquee in the gardens. The guests then gradually dispersed.

